# Investigation on natural resources and species conservation of *Ophiocordyceps sinensis*, the famous medicinal fungus endemic to the Tibetan Plateau

**DOI:** 10.1007/s13238-017-0406-6

**Published:** 2017-04-10

**Authors:** Wenjing Wang, Ke Wang, Xiaoliang Wang, Ruiheng Yang, Yi Li, Yijian Yao

**Affiliations:** 1 Institute of Microbiology, Chinese Academy of Sciences, Beijing, 100101 China; 2 University of Chinese Academy of Sciences, Beijing, 100049 China; 3 Institute of Edible Fungi, Shanghai Academy of Agricultural Sciences, Shanghai, 201403 China

**Figure 1:**
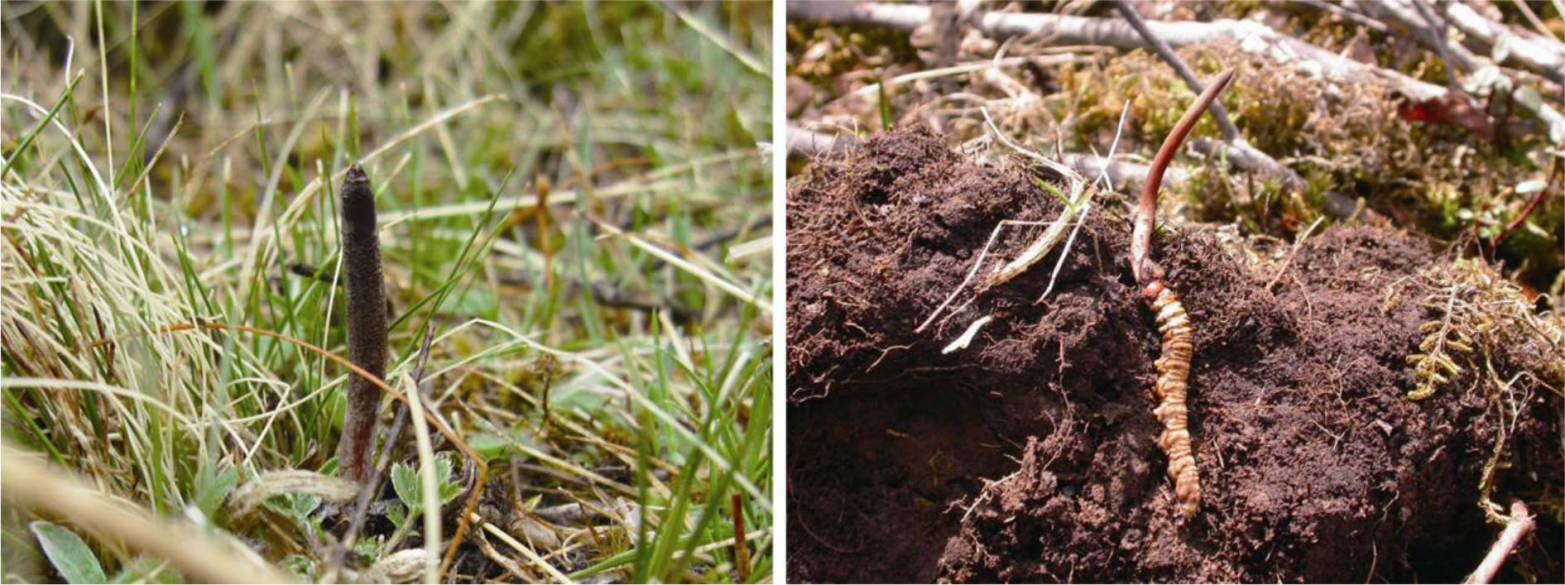
***Ophiocordyceps sinensis*** **in natural habitat (left) and freshly collected after cleaning (right)**

*Ophiocordyceps sinensis* (Berk.) G.H. Sung, J.M. Sung, Hywel-Jones & Spatafora, an entomogenous fungus endemic to the Tibetan Plateau, is one of the best known medicinal fungi in China and around the world. It has been documented in literature for hundreds of years and officially classified as a drug in the Chinese Pharmacopeia (Committee of Pharmacopeia, Chinese Ministry of Health, 2005). The whole fungus, including a stroma emerged above the ground from an underground sclerotium covered by the exoskeleton of a moth larva (Fig. 1), is harvested for medicinal uses, usually as tonic to strengthen the body system and to regain energy after a serious illness (Pegler et al., 1994) but now for many other purposes.

**Figure 2:**
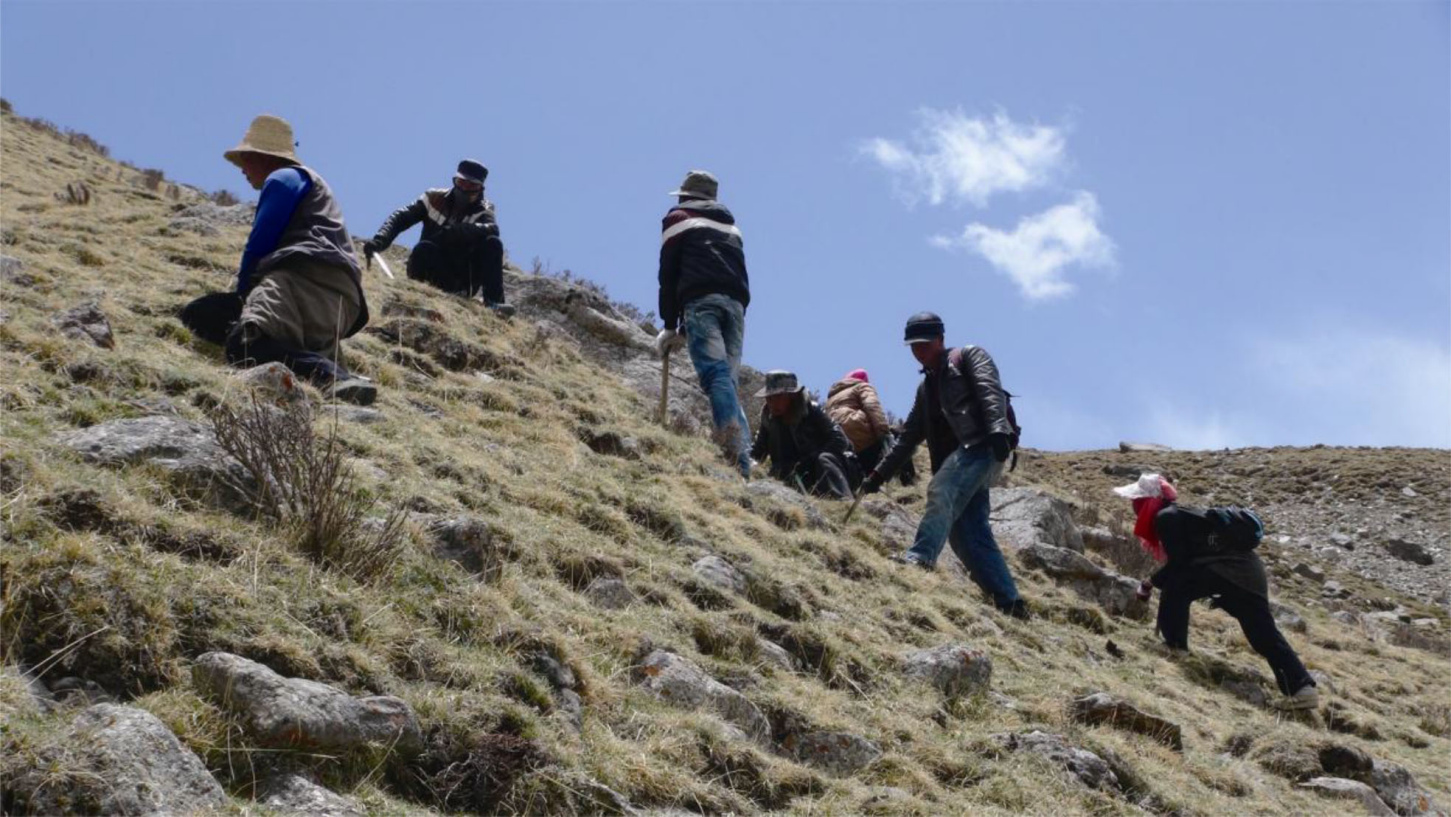
**Collectors in Golog Tibetan Autonomous Prefecture, Qinghai Province, China, photoed on June 4, 2014**

*Ophiocordyceps sinensis* occurs in alpine meadow and shrub habitats from an altitude of 3,000 m up to the snow-line (Li et al., 2011). Since *O*. *sinensis* is now sold at a price much higher than the gold, thousands and thousands of collectors are crowding onto the Tibetan Plateau to plunder this valuable resource during late spring and summer every year (Fig. 2). Due to its strict host-specificity, confined geographic distribution, climate changes and over exploitation by human, *O. sinensis* has suffered from a sharp decrease in natural production in recent decades. The annual production was recorded up to 100 tons in 1950s and ranged from 50–80 tons in 1960s but sharply decreased to 5–15 tons in 1990s (Li, 1998). In 1999, the fungus was listed as an endangered species under the second class of state protection (State Forestry Administration and Ministry of Agriculture, 1999).

Investigation on the resource of *O*. *sinensis* in China started from 1952, mainly focusing on its natural distribution. Since 2000, the information on distribution of the fungus became widely available online. Most of the materials online were short articles with a list of localities, often at county level or above, without any analysis. There were not many reports based on fieldwork to determine the distribution range of the species. Till 2011, the distribution of *O*. *sinensis* at the county level was clarified based on extensive fieldwork during the years 2000–2010 by the research group from the Institute of Microbiology, Chinese Academy of Sciences, on specimens preserved in HMAS (Fangrium, Institute of Microbiology, Chinese Academy of Sciences) and HKAS (Herbarium of Cryptograms, Kunming Institute of Botany, Chinese Academy of Sciences) and on examination of literature of more than 3600 publications. A total of 203 localities at county level for *O*. *sinensis* were recorded and analyzed. Among them, 106 were considered as confirmed distribution sites, 65 as possible distribution sites, 29 as excluded distribution sites, and three as suspicious distribution sites for the fungus (Li et al., 2011). In addition, the host insect species of the fungus was also summarized based upon an extensive literature survey. A total of 91 insect names spanning 13 genera of Hepialidae were gathered. Fifty-seven species were considered as recognizable potential host insects of *O*. *sinensis*, whilst eight as indeterminate hosts and 26 as non-hosts (Wang and Yao, 2011).

Genetic divergence in *O*. *sinensis* has been observed using different molecular markers and the southern populations were found more diverse than that of northern populations (Jiao, 2010). It is also speculated that *O*. *sinensis* spreads from a center of origin (the Nyingchi District) to southern regions and subsequently to northern areas (Zhang et al., 2009). Further, the correlation of genetic distances between *O*. *sinensis* and its host insects indicated that they coevolved through the history (Quan et al., 2014).

As one of the most valued living resources for local economy on the Tibetan Plateau and adjacent regions, further efforts should be devoted to learning more about the species conservation and resource management for this special fungus. So far, annual harvest, resource reservation, and quality comparison of *O*. *sinensis* in different geographic regions are less studied. Meanwhile, the natural distribution and the annual yield per unit area for this species are both dwindling based on feedbacks from local households, harvesters, and modeling predictions. The decline may result from the overexploitation, overgrazing and climate change, but there is no doubt that the changes in its distribution range and yield should be monitored by long-term field observation and statistical analyses.

Assessments of *O*. *sinensis* on its distribution and sustainable situation have been reported from Western Sichuan, Qinghai, and Northern Yunnan Provinces in China and from some areas in Nepal. The data were generally collected from households, harvesters and respondents by interviews, group discussions, informal communications, and personal field observations. The contribution to livelihoods and community development and the population status of *O*. *sinensis* were documented. However, there are no systematic management plans and national efforts to sustain these precious fungal resources. The extensive survey and the long-term field observation are important for successful management to protect natural resources of *O*. *sinensis* and to facilitate the improvement of the adaptive capacity to survive in the changing environment as revealed recently by Yan et al., (2017).

The extensive survey and long-term field observation for *O*. *sinensis* can be structured to target at: (i) to organize the survey of the species occurrence through administrative division with the support from the government from prefecture level down to every country, township and village, marking the production area to each mountain, finding the dates of emerging and terminating of the stroma, calculating annual yields based on real harvest; (ii) to determine the site for long-term field observation based on the habitat, fungal genetic background, transportation and living facilities at the location, to conduct the long-term observation with climate, soil and environment factors and also physical, chemical and biological characters, e.g., temperature, humidity, precipitation, organic matter, soil texture, pH, vegetation types, biomass, community structure, animals, soil microbes, and so on, and to monitor the occurrence of the fungus and its host insects, as well as the harvest grazing and human activities; (iii) to establish special natural reserves for the fungus and to carry out the management for sustainable uses of the fungal resources, including local laws and government regulations, time and methods of harvesting, measures for improvement of grassland for the fungus and its host insects, etc.; (iv) to construct the comprehensive database for recording and management of the fungus, its host insects, and the grassland for its survival and reproduction.

Along with market demands, *O*. *sinensis* is largely consumed in its dried form as a stroma emerged from a sclerotium which is covered by the exoskeleton of caterpillar larva. The maturity of the stroma is considered very important to the quality of the product. The immature specimens with stromata protruded from intact and solid sclerotium are much more welcomed than those with well grown or mature stromata because the nutritional reserves within stromata have been exhausted. To maintain the medicinal value, the stromata and sclerotia of *O*. *sinensis* are harvested before they start to produce and release the spores for reproduction. It is an urgent need to manage the natural resources of *O*. *sinensis* which have limited natural production but are so extensively harvested by human overexploitation.
